# Analysis of Clinical Features of Non-steroidal Anti-inflammatory Drugs Induced Kounis Syndrome

**DOI:** 10.3389/fcvm.2022.901522

**Published:** 2022-07-11

**Authors:** Chunjiang Wang, Weijin Fang, Liying Song, Zhenzhen Deng, Zuojun Li, Linli Sun

**Affiliations:** ^1^Department of Pharmacy, The Third Xiangya Hospital, Central South University, Changsha, China; ^2^Department of General Surgery, The Third Xiangya Hospital, Central South University, Changsha, China

**Keywords:** Kounis syndrome, anaphylaxis, coronary vasospasm, allergic reaction, non-steroidal anti-inflammatory drugs

## Abstract

**Background:**

Current knowledge of Kounis syndrome induced by non-steroidal anti-inflammatory drugs (NSAIDs) is based on case reports. This study aimed to investigate the clinical features of Kounis syndrome.

**Methods:**

Case reports of the NSAIDs-induced Kounis syndrome were analyzed by searching Chinese and English databases from 1 January 1950 to 31 January 2022.

**Results:**

The median age of the 45 included patients (28 women) was 51 years (20–80 years). NSAIDs that were the most frequently involved were diclofenac (26.7%, 12/45), metamizole (15.6%, 7/45), and aspirin (13.3%, 6/45). Kounis syndrome occurred mainly within 30 min after administration, with a maximum latency of 1 month. Chest pain (75.6%, 34/45), dyspnea (33.3%, 15/45), and allergic reactions (44.4%, 20/45) were the most common clinical manifestations. Thirty patients (66.7%) had an ST-segment elevation on the electrocardiogram. Echocardiogram and coronary angiography showed abnormalities in 21 patients (75%, 21/28) and 15 patients (37.5%, 15/40). Forty-four patients (97.8%) had a good prognosis after treatment with steroids, antihistamines, and vasodilators.

**Conclusion:**

The possibility of Kounis syndrome should be considered in the presence of coronary artery disease symptoms when taking NSAIDs. Kounis syndrome can be life-threatening. It is essential to identify and treat Kounis syndrome correctly.

## Introduction

Kounis syndrome is a rare acute coronary syndrome (ACS) caused by an allergic reaction that was first described as “anaphylactic angina syndrome” in 1991 (1). The main clinical signs and symptoms of Kounis syndrome are associated with allergic reactions accompanied by cardiac symptoms. Kounis syndrome can be induced by drugs, food, environmental exposures, and other conditions (2). Furthermore, Kounis syndrome causes coronary spasms and affects the cerebral and mesenteric arteries (3, 4).

The true incidence of Kounis syndrome is unknown. Many cases may be missed or underdiagnosed due to their variable presentation and the ignorance of the physician. Epidemiological studies are lacking to determine its prevalence. The unique clinical manifestations and treatment of Kounis syndrome have attracted clinical attention. Non-steroidal anti-inflammatory drugs (NSAIDs) are a class of drugs with antipyretic, analgesic, and anti-inflammatory effects. NSAIDs are associated with allergic reactions (5), and the incidence of hypersensitivity reactions is second only to antibiotics (6). Despite the widespread use of NSAIDs, NSAIDs-induced Kounis syndrome is uncommon.

Current knowledge of NSAIDs-induced Kounis syndrome is based on case reports. Furthermore, the syndrome is rarely recognized or reported in clinical practice due to incorrect diagnosis. This study aimed to investigate the clinical characteristics of NSAIDs-induced Kounis syndrome and provide evidence for clinical diagnosis and treatment.

## Materials and Methods

### Retrieval Strategy

We searched the Chinese and English databases from 1 January 1950, through 31 January 2022, including Wanfang, CNKI, VIP, PubMed, Embase, the Cochrane Library, and Web of science. The combination of subject headings and free texts was used for searching. English search terms included: NSAIDs, anaphylaxis, hypersensitivity, coronary vasospasm, cardiac arrest, allergy, Kounis syndrome, non-steroidal anti-inflammatory drugs, various approved non-steroidal anti-inflammatory drugs, myocardial infarction, acute coronary syndrome, and chest pain. We performed an initial evaluation of the titles and abstracts of the articles and read the full texts of all potentially eligible articles. References from studies were checked to identify additional eligible studies.

### Inclusion and Exclusion Criteria

Case reports and case series were included. Reviews, animal studies, mechanistic studies, and duplicate cases were excluded.

### Data Collection

Two authors independently extracted relevant clinical data according to self-designed tables, including patient country, gender, age, allergy history, past disease history, type and route of administration of NSAIDs, clinical symptoms, laboratory tests, imaging examination, treatment, and prognosis.

### Subtypes of Kounis Syndrome

Three variants of Kounis syndrome have been identified (7). The type I variant occurs in patients with structurally normal coronary arteries without cardiovascular risk factors. The acute release of inflammatory mediators induces coronary vasospasm, which may or may not result in acute myocardial infarction. The type II variant occurs in patients with preexisting coronary artery disease. The acute release of inflammatory mediators induces coronary vasospasm, leading to plaque rupture and myocardial infarction. The type III variant occurs in patients with a coronary artery stent. The release of inflammatory mediators can result in stent thrombosis. In this analysis, the type of Kounis syndrome was classified.

### Statistical Analysis

Statistical analysis was performed using SPSS 22.0. Descriptive analysis was performed on the extracted data. The measurement data are represented by the median value (minimum, maximum). Enumeration data are expressed as percentages.

## Results

### Identified Studies

**Figure 1:**
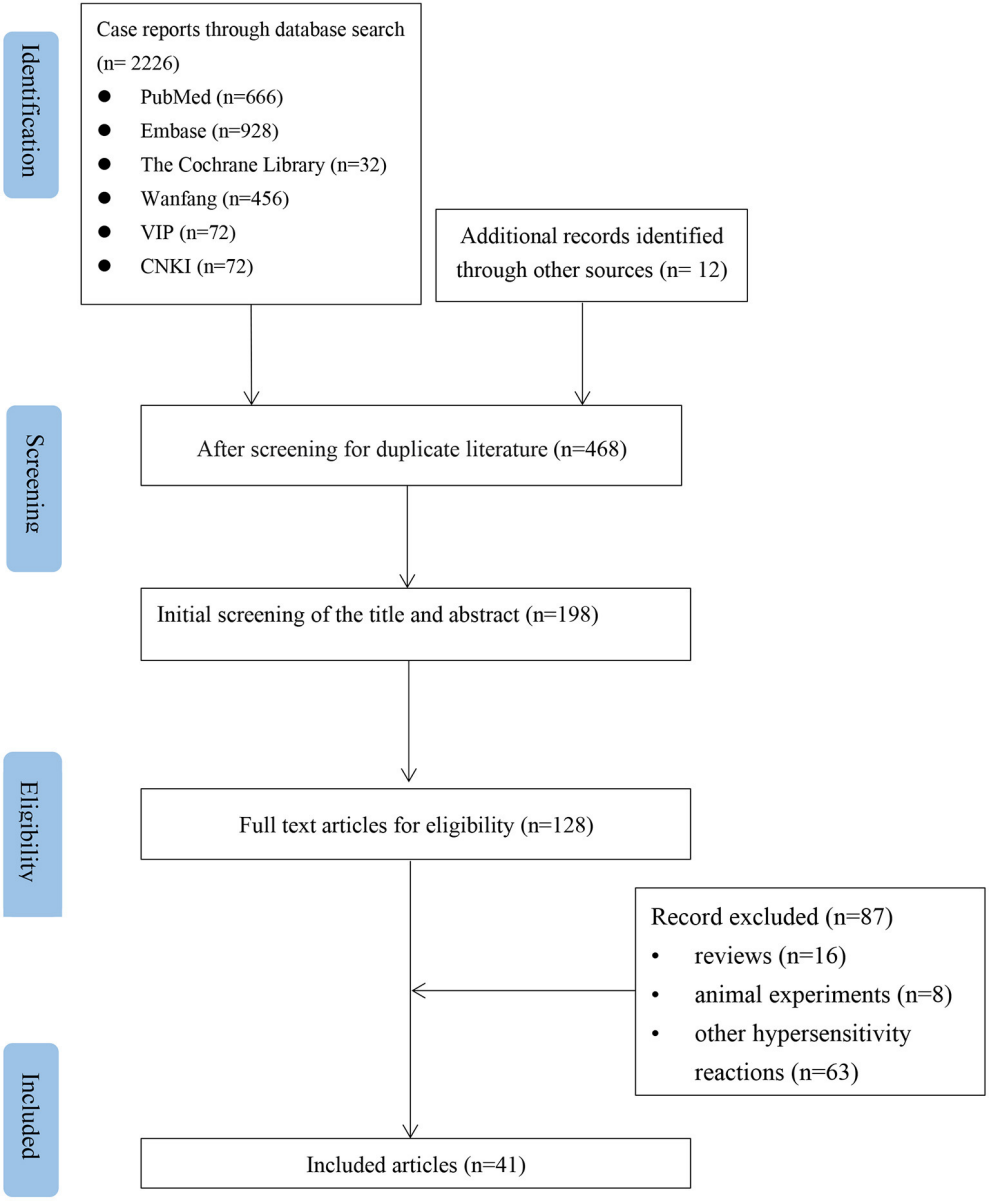
Flow chart of the study selection process for reported cases of NSAIDs-induced Kounis syndrome.

According to the inclusion and exclusion criteria, after independent screening by two authors, 41 articles were included, all of which were case reports. The literature screening is shown in Figure 1. The bias assessment of the case reports was evaluated using the National Institute for Clinical Excellence (NICE) quality scale. The quality of the included reports was low, with an overall score of 3–5.

### Basic Information

A total of 45 patients (28 women) were included, with a median age of 51 years (range 20–80) (Table 1). These patients were mainly from Asia (17.80%, 8/45) and Europe (68.9%, 31/45). The NSAIDs most commonly implicated were diclofenac (26.7%, 12/45), metamizole (15.6%, 7/45), aspirin (13.3%, 6/45), and ibuprofen (11.1%, 5/45). NSAIDs were used mainly to treat pain in 29 patients (82.9%). The most administration routes of NSAIDs were oral (52.5%, 21/40), intravenous (20.0%, 8/40), intramuscular (17.5%, 7/40), suppositories (5.0%, 2/40), intra-articular (2.5%, 1/40), and eye drops (2.5%, 1/40). Twenty patients (44.4%) had previous allergic diseases or reactions. Seventeen patients (37.8%) had cardiovascular diseases or cardiovascular risk factors.

**Table 1:** Basic information on NSAIDs-induced Kounis syndrome (*n* = 45).

**Parameter**		**Value**
Age	Years	51 (20,80)^b^
Sex	Male:female	17:28
Country	Turkey Spain USA Japan India Portugal Austria Morocco Netherlands Saudi Arabia UK Vietnam	20 (44.4%) 6 (13.3%) 4 (8.9%) 4 (8.9%) 3 (6.7%) 2 (4.4%) 1 (2.2%) 1 (2.2%) 1 (2.2%) 1 (2.2%) 1 (2.2%) 1 (2.2%)
Types of NSAIDs	Diclofenac Metamizole Aspirin Ibuprofen Dexketoprofen Naproxen Paracetamol Nimesulide Acemetacine Propyphenazone Celecoxib Etofenamate Indomethacin	12 (26.7%) 7 (15.6%) 6 (13.3%) 5 (11.1%) 3 (6.7%) 3 (6.7%) 3 (6.7%) 1 (2.2%) 1 (2.2%) 1 (2.2%) 1 (2.2%) 1 (2.2%) 1 (2.2%)
Indication (35)^a^	pain coronary artery disease upper respiratory tract infection fever	29 (82.9%) 2 (5.7%) 3 (8.6%) 1 (2.9%)
Route of administration (40)^a^	oral intravenous intramuscularly suppository intraarticular eye drops	21 (52.5%) 8 (20.0%) 7 (17.5%) 2 (5.0%) 1 (2.5%) 1 (2.5%)
Delay (31)^a^	Within 30 min 1h−24h 1 month	22 (71.0%) 8 (25.8%) 1 (3.2%)
Allergic conditions or history	drug allergy, asthma, seasonal allergic rhinitis, shaving foam, fruits	20 (44.4%)
Cardiovascular disease or risk factors (17)^a^	smoking hypertension diabetes coronary artery disease hyperlipidemia	13 (28.9%) 9 (20.0%) 4 (8.9%) 4 (8.9%) 3 (6.7%)

*NSAIDs, Nonsteroidal anti-inflammatory drugs*.

a *Represents the number of patients out of 21 in whom information regarding this particular parameter was provided*.

b *Median (minimum-maximum)*.

### Clinical Symptoms

The clinical symptoms of these patients are summarized in Table 2. Kounis syndrome occurred mainly within 30 min after administration, with a maximum latency of 1 month. Chest pain (75.6%, 34/45) and dyspnea (33.3%, 15/45) were the most common symptoms of the acute coronary syndrome. Other common symptoms were digestive symptoms (nausea, vomiting) in 15 patients (33.3%) and neurological symptoms (lightheadedness, syncope, unconsciousness) in 10 patients (22.2%). Thirty-three patients (73.3%) had allergic reactions manifested as itching (42.2%, 19/45), rash (40.0%, 18/45), erythema (20.0%, 9/45), and skin flushing (13.3%, 6/45). Hypotension occurred in 25 patients (69.4%, 25/36). Four patients (8.9%) had a cardiac arrest. Three patients (6.7%) had cardiogenic shock.

**Table 2:** Clinical symptoms and laboratory findings of NSAIDs-induced Kounis syndrome.

**Parameter**		**Value**
Clinical symptoms	Chest pain Dyspnea Nausea Vomiting Shortness Of Breath Sweating Lightheadedness Palpitations Syncope Unconscious Cyanosis Abdominal Discomfort Arm Pain Allergy Itching Rash Erythema Flushing Swelling	34 (75.6%) 15 (33.3%) 8 (17.8%) 7 (15.6%) 7 (15.6%) 6 (13.3%) 5 (11.1%) 5 (11.1%) 3 (6.7%) 2 (4.4%) 1 (2.2%) 1 (2.2%) 1 (2.2%) 33 (73.3%) 19 (42.2%) 18 (40.0%) 9 (20.0%) 6 (13.3%) 3 (6.7%)
Cardiac Arrest	Yes No	4 (8.9%) 41(91.1%)
Blood pressure (36)^a^	hypotension normal pressure	25 (69.4%) 11 (30.6%)
Troponin (38)^a^	elevated normal	32 (84.2%) 6 (15.8%)
Creatine kinase (25)^a^	elevated normal	15 (60.0%) 10 (40.0%)
Serum tryptase (10)^a^	elevated normal	7 (70.0%) 3 (30.0%)
IgE (12)^a^	elevated normal	10 (83.3%) 2 (16.7%)

*NSAIDs, Nonsteroidal anti-inflammatory drugs; IgE, Immunoglobulin E*.

a *Represents the number of patients out of 21 in whom information regarding this particular parameter was provided*.

### Laboratory Test

Laboratory tests are summarized in Table 2. Troponin was elevated in 32 patients (84.2%, 32/38), creatine kinase was elevated in 15 patients (60.0%, 15/25), serum tryptase was elevated in 7 patients (70.0%, 7/10), and immunoglobulin E (IgE) was elevated in 10 patients (83.3%, 10/12). Two patients had positive skin prick tests.

### Imaging Examination

The imaging results are summarized in Table 3. The most common presentations of electrocardiogram (ECG) were ST-elevation (66.7%, 30/45) and ST-elevation and ST-depression (20.0%, 9/45). Echocardiography in 28 patients showed hypokinesia (39.3%, 11/28), normal (35.7%, 10/28), and reduced ejection fraction (21.4%, 6/28). Coronary angiography showed that 13 patients (32.5%, 13/40) had occlusion, stenosis, and plaques, and two patients (5%, 2/40) had vasospasm. The main coronary arteries involved were the right coronary artery (RCA) and the left anterior descending artery (LAD). The categories of Kounis syndrome were type I variant (73.3%, 33/45), type II variant (24.4%, 11/45), and type III variant (2.2%, 1/45).

**Table 3:** Imaging of NSAIDs-induced Kounis syndrome.

**Parameter**		**Value**
Electrocardiograph (45)^a^	ST elevation ST elevation and ST depression Atrioventricular block ST depression T-wave inversion Atrial fibrillation Negative T wave Prolonged QT interval	30 (66.7%) 9 (20.0%) 5 (11.1%) 3 (6.7%) 3 (6.7%) 2 (4.4%) 1 (2.2%) 1 (2.2%)
Echocardiography (28)^a^	Hypokinesia Normal Systolic dysfunction RWMA Preserved ejection fraction Decreased ejection fraction	11(39.3%) 10 (35.7%) 2 (7.1%) 2 (7.1%) 3 (10.7%) 6 (21.4%)
Coronary angiography (40)^a^	Normal Occlusion Stenosis Plaques Vasospasm	26 (65.0%) 9 (22.5%) 2 (5.0%) 2 (5.0%) 2 (5.0%)
Involved arteries (14)^a^	LCX LCA RCA LAD	1 (7.1%) 2 (14.3%) 6 (42.9%) 9 (64.3%)
Kounis syndrome type^a^	Type I variants Type II variants Type III variants	33 (73.3%) 11 (24.4%) 1 (2.2%)

*RWMA, regional wall motion abnormalities; RCA, right coronary artery; LCX, left circumflex artery; LAD, left anterior descending artery*.

a *Represents the number of patients out of 21 in whom information regarding this particular parameter was provided*.

### Treatment and Prognosis

NSAIDs were discontinued in all patients after developing Kounis syndrome (Table 4). Twenty-seven patients (60.0%) received corticosteroids, 26 (57.8%) received antihistamines, and 13 (28.9%) received antiplatelet drugs. Twelve patients (26.7%) received vasodilators including calcium channel blockers (17.8%, 8/45), nitrate (15.6%, 7/45), beta-blockers (4.4%, 2/45), nicorandil (4.4%, 2/45), and ranolazine (2.2%, 1/45). Five patients (11.1%) received intracoronary nitroglycerin and six (13.3%) received heparin. Eight patients (17.8%) underwent percutaneous coronary intervention (PCI). Ultimately, 44 patients (97.8%) had improvement in symptoms or recovered, and one patient (2.2%) died.

**Table 4:** Treatment and prognosis of NSAIDs-induced Kounis syndrome.

**Parameter**		**Value**
Treatment	Discontinued Corticosteroid Antihistamine Antiplatelet Adrenaline Calcium channel blocker Intracoronary nitroglycerin Nitrate Heparin Morphine Beta blocker Ranolazine Statin nicorandil Percutaneous coronary intervention	45 (100%) 27 (60.0%) 26 (57.8%) 13 (28.9%) 8 (17.8%) 8 (17.8%) 5 (11.1%) 7 (15.6%) 6 (13.3%) 3 (6.7%) 2 (4.4%) 1 (2.2%) 2 (4.4%) 2 (4.4%) 8 (17.8%)
Prognosis	Improve or recover Die	44 (97.8%) 1 (2.2%)

## Discussion

NSAIDs are reversible inhibitors of cyclooxygenase (COX)-1 and COX-2 with varying degrees of selectivity (8) and can be divided into five categories according to the degree of inhibition of COX-1 and COX-2 (9). NSAIDs have long been associated with an increased risk of vascular events (10). NSAIDs can alter the balance of thromboxane-prostacyclin, leading to vasospasm and the formation of a platelet thrombus. The cardiovascular risk may be drug-specific, and further studies are needed to define cardiovascular risk related to NSAIDs (11).

However, the cardiovascular hazard of NSAIDs was driven primarily by the increase in the risk for non-ST-segment elevation. In contrast, NSAIDs did not increase ST-segment elevation myocardial infarction (12). Among the 51 cases of Kounis syndrome reported to the International Agency for Pharmacovigilance (VigiBase™) between 2010 and 2014, most of the cases occurred in the United States. NSAIDs were the most common trigger drugs (13).

In our analysis, NSAIDs-induced Kounis syndrome was more common in southern Europe, especially Turkey and Spain. Type I Kounis syndrome was the predominant type and usually occurred within 30 min. One case of Kounis syndrome occurred 1 month after taking NSAIDs. The probabilities of Kounis syndrome caused by NSAIDs are different, which can be explained by the immune mechanisms of the NSAIDs. Non-allergic NSAIDs reactions are allergy-like reactions that are not immunologically mediated. These reactions are thought to occur mainly due to the inhibition of COX-1 enzymes. Non-allergic NSAIDs reactions are known to be cross-reactive. Immunologically mediated NSAIDs reactions are based on immunoglobulin E (IgE) or T cell response. These reactions do not depend on COX-1 inhibition and can be induced by a single NSAIDs or by a class of NSAIDs with similar chemical structures (14).

The diagnosis of Kounis syndrome is based on clinical signs and symptoms, laboratory tests, ECG, echocardiogram, and coronary angiography (2). Risk factors for Kounis syndrome include previous allergies, hypertension, smoking, diabetes, and hyperlipidemia (7). For patients with suspected Kounis syndrome, a careful review of the clinical history is warranted, including medications and allergy history. In our analysis, patients with NSAIDs-induced Kounis syndrome were predominantly female (62%), contrary to Abdelghany et al. (15). In our study, 44.4% of the patients had a history of allergies or allergic conditions, higher than previously reported (25.1%) (15). ECG usually shows ST-T changes suggesting ischemia, with ST-elevation being the most common finding. Cardiac catheterization may show coronary vasospasm or stenosis. Our study showed that LAD was the culprit artery in >64% of cases, followed by RCA.

Kounis syndrome is not only a single organ arterial disease but also a complex multiorgan disease that can affect the skin, respiratory, and vascular systems (16). Signs and symptoms can be variable, depending on the organ systems. In addition to cardiac manifestations, Kounis syndrome involves the skin or mucosal surfaces (e.g., pruritus, rash, erythema), gastrointestinal system (e.g., diarrhea, vomiting), respiratory system (e.g., shortness of breath, dyspnea), cardiovascular system (e.g., hypotension, palpitations), and nervous system (e.g., unconsciousness, syncope). The severity of Kounis syndrome can range from mild angina with urticaria and pruritus to cardiogenic shock.

Cardiac tissue contains abundant mast cells (17). Infiltration of activated mast cells into plaque erosion or rupture areas is a common pathway between allergic and non-allergic coronary events (18). The burden of cardiac mast cells in coronary plaques in patients with heart disease is 200 times greater than in the coronary arteries in healthy individuals (18). Kounis syndrome is caused by the action of pro-inflammatory mediators released in abundance by mast cells in cardiac tissue, coronary arteries, and plaques. These inflammatory mediators (for example, histamine, neutral proteases, arachidonic acid products, platelet-activating factor, and heparin) lead to peripheral vasodilation, decreased blood pressure and coronary blood flow, coronary spasm, atherosclerotic plaque erosion rupture, and coronary stent thrombosis (19–22).

The treatment of Kounis syndrome is highly challenging. At present, treatment is empirical with no professional guidelines. It is necessary to treat the symptoms of the heart and allergies simultaneously. Symptoms can be eliminated in patients with type I variants after antiallergic treatment. Antiallergic treatment can be performed with intravenous corticosteroids (e.g., hydrocortisone) and H1 and H2 receptor antagonists (e.g., diphenhydramine and ranitidine) (23, 24). Administration of vasodilators, such as calcium channel blockers and nitrates, can eliminate vasospasms caused by hypersensitivity reactions (25). For patients with type II variants, acute coronary events must be managed along with anti-allergic therapy with corticosteroids and antihistamines (2). For patients with type III variants, a critical myocardial infarction protocol and emergency thrombus aspiration should be performed, followed by histological examination of the aspirated material and staining for eosinophils and mast cells (26). Epinephrine should be used with caution in Kounis syndrome because it aggravates ischemia, prolongs the QT interval, and causes coronary spasms or arrhythmias (27). Beta-blockers can exacerbate coronary spasms due to the lack of antagonism of α-adrenergic receptors (27).

Stabilizing mast cells and preventing the release of inflammatory mediators may represent a novel therapeutic strategy for Kounis syndrome (23, 28–30). Agents that target stem cell factors are essential for mast cell development, proliferation, survival, adhesion, and homing. These agents include mediator antagonists, inhibitors of mediator biosynthesis, leukotriene antagonists, mediator receptor blockers (sodium nedocromil, sodium cromoglycate, ketotifen, lodoxamide), humanized IgG1 monoclonal antibodies, and other natural molecules that interfere with mast cell stabilization and prevent the release of mast cell contents (31).

Various factors affect the prognosis of Kounis syndrome, including comorbidity, sensitivity, the site of the antibody-antigen reaction, allergen entrance, the allergen concentration, number of allergens the patient is exposed to, and the magnitude of the initial allergic response (21, 32). Type I Kounis syndrome has a more favorable prognosis than the other two variants (15). In our analysis, serious complications of NSAIDs-induced Kounis syndrome were rare, with cardiogenic shock at 6.7% and cardiac arrest at 8.9%. Only one patient with dexketoprofen-induced type I Kounis syndrome died from cardiac arrest (33). The remaining 97.8% of the patients recovered or had symptoms improved after appropriate treatment without any associated long-term sequelae.

## Conclusion

Kounis syndrome is a rare adverse effect of NSAIDs. The risk of myocardial infarction must be considered when prescribing NSAIDs. Physicians should promptly recognize Kounis syndrome and treat patients with antihistamines, steroids, and calcium channel blockers. Patients may have a good prognosis when appropriate and timely treatment is administered.

## Data Availability Statement

The raw data supporting the conclusions of this article will be made available by the authors, without undue reservation.

## Author Contributions

LSu and CW conceived of the presented idea. CW, WF, LSo, ZD, ZL, and LSu wrote the manuscript. All authors discussed the results and contributed to the final manuscript. All authors contributed to the article and approved the submitted version.

## Funding

This study was funded by the Hunan Provincial Natural Science Foundation (No. 2021JJ30992).

## Conflict of Interest

The authors declare that the research was conducted in the absence of any commercial or financial relationships that could be construed as a potential conflict of interest. The reviewer YJ declared a shared affiliation with the author(s) to the handling editor at the time of review.

## Publisher's Note

All claims expressed in this article are solely those of the authors and do not necessarily represent those of their affiliated organizations, or those of the publisher, the editors and the reviewers. Any product that may be evaluated in this article, or claim that may be made by its manufacturer, is not guaranteed or endorsed by the publisher.
